# Computational identification of potential multitarget treatments for ameliorating the adverse effects of amyloid-β on synaptic plasticity

**DOI:** 10.3389/fphar.2014.00085

**Published:** 2014-05-08

**Authors:** Thomas J. Anastasio

**Affiliations:** Department of Molecular and Integrative Physiology, Beckman Institute for Advanced Science and Technology, University of Illinois at Urbana-ChampaignUrbana, IL, USA

**Keywords:** Alzheimer disease, computational model, formal methods, drug targets, multidrug therapy

## Abstract

The leading hypothesis on Alzheimer Disease (AD) is that it is caused by buildup of the peptide amyloid-β (Aβ), which initially causes dysregulation of synaptic plasticity and eventually causes destruction of synapses and neurons. Pharmacological efforts to limit Aβ buildup have proven ineffective, and this raises the twin challenges of understanding the adverse effects of Aβ on synapses and of suggesting pharmacological means to prevent them. The purpose of this paper is to initiate a computational approach to understanding the dysregulation by Aβ of synaptic plasticity and to offer suggestions whereby combinations of various chemical compounds could be arrayed against it. This data-driven approach confronts the complexity of synaptic plasticity by representing findings from the literature in a course-grained manner, and focuses on understanding the aggregate behavior of many molecular interactions. The same set of interactions is modeled by two different computer programs, each written using a different programming modality: one imperative, the other declarative. Both programs compute the same results over an extensive test battery, providing an essential crosscheck. Then the imperative program is used for the computationally intensive purpose of determining the effects on the model of every combination of ten different compounds, while the declarative program is used to analyze model behavior using temporal logic. Together these two model implementations offer new insights into the mechanisms by which Aβ dysregulates synaptic plasticity and suggest many drug combinations that potentially may reduce or prevent it.

## Introduction

Alzheimer Disease (AD) is the most prevalent form of dementia (Whitehouse et al., 2000). Despite decades of intensive basic and clinical investigation, an effective treatment for AD is still lacking. Part of the difficulty in developing treatments for AD may be the sheer complexity of the pathological processes that cause it. The earliest signs of AD are cognitive impairments including deficits in memory formation and storage that are caused by disruption of the processes of synaptic plasticity, which are complex in their own right. One avenue toward understanding these complex processes is to represent, simulate, and analyze them using “process algebra,” a computational technique that falls under the umbrella of formal methods in computer science (Monin and Hinchey, 2003). The purpose of this study is to develop an initial computational framework for understanding how various chemical compounds might aid memory in AD by using formal methods to simulate the deficits in synaptic plasticity that accompany the disorder.

The model is based on the amyloid hypothesis, which posits that AD results from the buildup beyond normative levels of the peptide amyloid-β (Aβ). The amyloid hypothesis has been the predominant theory of AD etiology for the past two decades (Hardy and Selkoe, 2002). Doubts have surfaced concerning whether Aβ is the ultimate cause of AD in humans (Lee et al., 2004; Castellani et al., 2009; Mullane and Williams, 2013; Roher et al., 2013; Tayeb et al., 2013), but recent neuropathological research shows that individuals who have high levels of Aβ postmortem, but do not have soluble forms of Aβ localized to synapses, did not have prior cognitive impairment (Bjorklund et al., 2012). This finding is consistent with the results of studies on non-human animals showing that high Aβ levels cause abnormalities in synaptic plasticity. *In vivo*, intracerebroventricular administration of Aβ peptides in rats causes deficits in experimentally induced synaptic plasticity (Freir et al., 2001), while transgenic mice that overexpress Aβ show deficits in learning and memory (Hsiao et al., 1996; Chapman et al., 1999; Koistinaho et al., 2001; Howlett et al., 2004). The literature documenting the adverse effects on synaptic plasticity of soluble Aβ applied to synapses *in vitro* provides some of the data for this formal-methods model of the effects of Aβ on synaptic plasticity (see subsection Experimental Data on Aβ Effects on Synaptic Plasticity).

Obviously, prevention of Aβ overproduction would eliminate the adverse effects of Aβ on synaptic plasticity, and these adverse effects have indeed been reversed in transgenic mouse AD models in which Aβ overproduction was reduced pharmacologically (Klyubin et al., 2005; Ding et al., 2008; Klyubin et al., 2008; Peng et al., 2010; Townsend et al., 2010; Balducci et al., 2011; Medina et al., 2011). Unfortunately, the development of clinically safe pharmacological means to reduce Aβ in humans remains elusive (Cho and Kim, 2011; Schenk et al., 2012), and it is therefore of interest to explore ways to ameliorate the effects of Aβ on synaptic plasticity. The Alzheimer Research Forum currently lists over 80 chemical compounds at various stages of clinical evaluation for AD treatment (http://www.alzforum.org/therapeutics), and many of these aim to improve synaptic plasticity. The possible benefits for synaptic plasticity of ten compounds, administered alone or in combination, will be explored computationally using the formal-methods model introduced here.

In computer science, formal methods are computational methods for specifying and analyzing systems (Monin and Hinchey, 2003). In practice, formal methods are implemented by creating a model of a system that takes the form of a computer program written in a declarative programming language. Whereas the more conventional, imperative programming languages are designed for efficient computation and facilitate intensive simulation of system behavior under many different circumstances, declarative languages are designed to facilitate analysis of selected system behaviors via temporal logic (Huth and Ryan, 2004). Essentially, imperative programs are used to compute quickly and without keeping track of the sequence of computations that gave rise to the result. In contrast, declarative programs are slower but they do keep track of the sequences of computations they execute, and these sequences can later be searched for specific system states, or queried for evaluation of temporal-logic properties such as whether or not a specific state eventually occurs, never occurs, is always present, or present but only after the occurrence of some other specific state, and so on. The advantages of declarative programming for modeling and analysis of biological systems have been recognized, and early applications have appeared (Fisher and Henzinger, 2007). The declarative language used here is called Maude (Clavel et al., 2007). Maude has already been applied in modeling biological systems (Eker et al., 2002; Talcott, 2008). Applications of Maude include recent models of AD etiology (Anastasio, 2011, 2013). These previous publications can be consulted for more detailed explanations of the declarative programming approach to understanding biological systems.

The model described here is similar in form to the recent models of AD etiology (Anastasio, 2011, 2013) in that it is data-driven. As such, experimental findings as reported in the literature are represented as declarations in a Maude program (a.k.a. specification). Like the previous AD models, this model is coarse grained in that the details of specific molecular interactions are omitted. Only the basic, experimentally observed facts are retained. Previous authors have noted the huge variability in the experimental preparations that have been used to study synaptic plasticity, including those used to study the effects on it of Aβ (Nistico et al., 2012). The model developed here does not endeavor to represent this multitude of experimental configurations. Instead it adopts a generic framework, and suggests that experiments on synaptic plasticity in general, and on pharmacological modification of plasticity in specific, would be best done using a standard preparation (see also Discussion). The goal is to gain a high-level perspective on the interactions occurring within the system of many molecules that underlies normal synaptic plasticity, and on how that system is dysregulated by Aβ.

In addition to the declarative Maude specification, the same system of molecular interactions is also represented in a program written in MATLAB®, an imperative language. Both programs behave identically over an extensive test battery, and this crosscheck provides assurance that overall model behavior is consistent with basic findings on synaptic plasticity and that the results are not corrupted by programming error. The Maude version is used to analyze the performance of the model with or without Aβ but in the absence of any pharmacological intervention. Then the MATLAB version is used to screen for efficacy every one of the 1024 possible combinations of ten compounds that have been shown, by themselves, to ameliorate the adverse effects of Aβ on synaptic plasticity. The Maude version is used to analyze the mechanisms by which the 20 most efficacious combinations might actually work. The results provide new insights into how Aβ dysregulates the system of molecular interactions that underlies synaptic plasticity, and offers predictions concerning the possible ameliorative effects of 20 combinations of known compounds, which could be experimentally tested using a standardized preparation. The main benefits of the approach are that it represents many findings from the literature and shows what they imply in the aggregate, and that it provides an initial but rigorous means to discover possible drug combinations that improve outcomes over any single-drug therapy.

## Methods

### Experimental data on basic mechanisms of LTP and LTD

**Figure 1:**
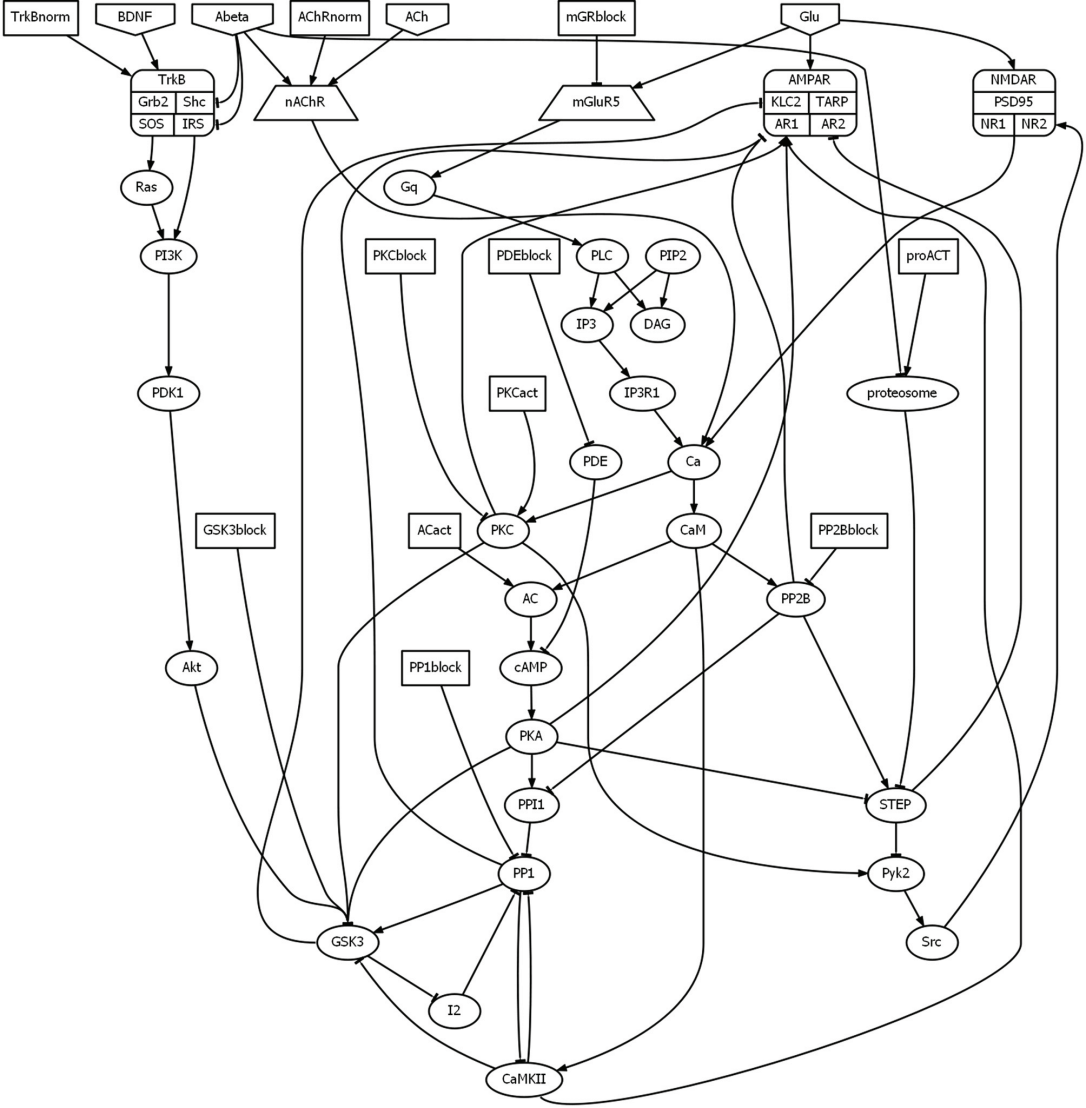
**Schematic of the data-driven model of the effects of Aβ on synaptic plasticity**. The diagram depicts the influences that each element has on the other elements in the model. Arrowheads and tees represent positive and negative influences, respectively. The diagram was drawn using Graphviz software. The list of abbreviations is provided in Table 1.

The two forms of synaptic plasticity most relevant to AD neuropathology are long-term potentiation (LTP) and long-term depression (LTD), which are long-lasting increases and decreases in synaptic strength, respectively. The two brain regions most implicated in AD neuropathology are hippocampus and cerebral cortex (Eustache et al., 2000), and the *in vitro* studies on Aβ effects on LTP and LTD have been carried out using brain slices or cultures containing neurons from those two regions. The basic features of the molecular interactions that underlie LTP and LTD in hippocampus, frontal cortex, and other brain regions have been reviewed extensively (Malenka and Bear, 2004; Citri and Malenka, 2008; Stanton et al., 2010). For reasons of manageability and tractability of this initial model, it focuses on early, postsynaptic mechanisms of LTP and LTD that do not require new protein synthesis. The molecular interactions that mediate these mechanisms are diagrammed in Figure 1.

The neurotransmitter mediating transmission at most excitatory synapses in hippocampus and cortex is glutamate (Glu), and the receptor responsible for postsynaptic depolarization is the α-amino-3-hydroxy-5-methyl-4-isoxazole propionic acid receptor (AMPAR). Basically, changes in synaptic strength due to LTP or LTD involve increases or decreases, respectively, in the ionic conductance and/or presence in the postsynaptic density of AMPARs. The best known forms of LTP and LTD are triggered by activation by Glu of *N*-methyl-D-aspartate receptors (NMDARs). This requires concurrent depolarization of the postsynaptic terminal, which is usually a dendritic spine. Basically, sufficient depolarization makes it possible for Glu to activate NMDARs, which causes calcium ions (Ca) to flow into the postsynaptic spine. An intense, brief (several seconds) Ca increase leads to LTP while a more moderate, prolonged (several minutes) Ca increase leads to LTD (Yang et al., 1999).

Under natural circumstances, postsynaptic terminal depolarization would be produced by presynaptic activity and release of Glu onto spine AMPARs, which would cause them to allow sodium and potassium (but not calcium) ions to enter the spine. Experimentally, intense/brief or moderate/prolonged stimulation of presynaptic axons leads to LTP or LTD, respectively. AMPARs and NMDARs are colocalized on the postsynaptic density of dendritic spines via structural proteins such as postsynaptic density 95 (PSD95), and their activation leads to increases within the spine in cation concentration (sodium, potassium, and calcium, with calcium flowing through NMDARs only). Increased intra-spine Ca initiates an array of signal transduction mechanisms that contribute to the production of LTP or LTD. Essentially, the increases or decreases in AMPAR tone associated with LTP or LTD are driven by signal transductions that result in the activation of kinases or phosphatases, respectively.

Central to NMDAR-mediated LTP production is the kinase calcium/calmodulin-dependent protein kinase II (CaMKII), which is activated by Ca through calmodulin (CaM) (Lisman et al., 2012). Activated CaMKII potentiates the synapse by phosphorylating the AR1 subunit of the AMPAR receptor. This leads both to increased AMPAR ionic conductance and to increased AMPAR insertion into and retention by the postsynaptic density. The exocytotic process involves structural proteins known as transmembrane AMPAR regulatory proteins (TARPs). Other kinases also contribute to LTP, including protein kinase A (PKA), which is activated by cyclic adenosine monophosphate (cAMP) produced by adenylyl cyclase (AC), which itself is activated by Ca via CaM (Ferguson and Storm, 2004). PKA boosts CaMKII by activating phosphoprotein inhibitor 1 (PPI1), which inactivates protein phosphatase 1 (PP1). Because PP1 and CaMKII are mutually inhibitory (Strack et al., 1997), PP1 inactivation promotes CaMKII activation. Protein kinase C (PKC) and sarcoma (Src) kinase are also involved in LTP production because PKC activates Src via protein tyrosine kinase 2 (Pyk2) (Dikic et al., 1996), and Src enhances NMDAR function by phosphorylating the NR2 subunit (Huang et al., 2001). Also, both PKA and PKC phosphorylate AR1, leading to increased AMPAR conductance and/or insertion/retention. The kinases CaMKII, PKA, and PKC inactivate glycogen synthase kinase-3 (GSK3) (Li et al., 2000; Espada et al., 2009; Song et al., 2010), and this further promotes LTP because it removes an important contribution to LTD (see below in this subsection).

Intense presynaptic stimulation also causes secretion of brain-derived neurotrophic factor (BDNF), which binds to the tyrosine kinase B (TrkB) receptor and contributes to LTP production (Minichiello, 2009; Edelmann et al., 2014). Neurotrophin 4 (NT4), which also binds to TrkB, may play a similar role (Xie et al., 2000). BDNF-mediated LTP is associated with activation of protein kinase B (PKB, a.k.a. Akt) by phosphoinositide-3-kinase (PI3K) via phosphoinositide-dependent kinase-1 (PDK1) (Chan et al., 1999; Kelly and Lynch, 2000; Racaniello et al., 2010). Binding of BDNF to TrkB activates the insulin receptor substrate (IRS), and IRS activates PI3K (Yamada et al., 1997). Also, BDNF binding to TrkB activates the Src homology protein Shc and the Son of sevenless (SOS) protein, and the activations of IRS, Shc, and SOS by TrkB are all regulated by growth factor receptor bound-2 (Grb2) (Buday and Downward, 1993; Rozakis-Adcock et al., 1993; Skolnik et al., 1993). The activation of SOS may also require Shc (Minichiello, 2009). SOS then activates the Rat sarcoma kinase (Ras) (Rozakis-Adcock et al., 1993), and Ras activates PI3K (Rodriguez-Viciana et al., 1994). Thus, the cascade following BDNF binding to TrkB activates PI3K via two pathways. Once activated, Akt promotes inactivation of GSK3 (Cross et al., 1995; Peineau et al., 2007). As for CaMKII, PKA, and PKC, Akt promotes LTP by suppressing GSK3 because it removes an important contribution to LTD (see also below in this subsection).

Central to NMDAR-mediated LTD production is activation by CaM of protein phosphatase 2B (PP2B, a.k.a. calcineurin), which activates PP1 by inactivating PPI1 (Mulkey et al., 1994). The CaM affinity of PP2B is higher than that of CaMKII or AC (Kim et al., 2010). Thus low levels of Ca lead to activation of the phosphatases that drive LTD while high levels of Ca are required to activate the kinases that drive LTP. Phosphatases PP2B and PP1 depress the synapse by dephosphorylating the AR1 subunit of the AMPAR receptor, leading to decreased AMPAR ionic conductance and to increased AMPAR release and removal from the postsynaptic density. PP1 also contributes to LTD by inhibiting CaMKII, which is a kinase central to LTP production (see above in this subsection). PP1 further contributes to LTD by activating GSK3, a kinase that is actually activated by dephosphorylation (Peineau et al., 2007). GSK3 in turn promotes PP1 activation through inhibitory phosphorylation of inhibitor-2 (I2), which inhibits PP1 (Szatmari et al., 2005). GSK3 contributes to LTD production by phosphorylating kinesin light chain-2 (KLC2), which causes KLC2 to release the AR1 subunit leading to removal of AMPARs from the postsynaptic density (Du et al., 2010). Lithium (Li) can suppress LTD production by inactivating GSK3 (Peineau et al., 2007).

PP2B further contributes to LTD by activating striatal-enriched protein tyrosine phosphatase (STEP) (Snyder et al., 2005), and STEP drives LTD in several ways. STEP is most likely the phosphatase that dephosphorylates the AR2 subunit, leading to AMPAR removal from the postsynaptic density (Moult et al., 2006; Zhang et al., 2008). STEP also inactivates Src by inactivating Pyk2 (Xu et al., 2012). Since Src enhances NMDAR conductance, STEP reduces Ca flow through NMDARs by this pathway. STEP is inactivated by PKA (Blanco-Aparicio et al., 1999), which may be a critical interaction (see Results and Discussion).

Another mechanism by which Glu can induce LTD is through activation of class I metabotropic glutamate receptors (mGluRs), of which mGluR5 predominates in hippocampus and cortex (Anwyl, 2006). This form of LTD can also be evoked using the class I mGluR agonist dihydroxyphenylglycine (DHPG) (Huang and Hsu, 2006). Through coupling via the G-protein Gqα, activation of mGluR5 leads to activation of phospholipase C (PLCγ), which hydrolyses phosphatidylinositol bisphosphate (PIP2) yielding inositol trisphosphate (IP3) and diacyl glycerol (DAG). IP3 then activates its intracellular receptor IP3R1, which leads to Ca release from the smooth endoplasmic reticulum. This Ca could also lead via CaM to activation of phosphatases, as for NMDAR-mediated LTD. Interestingly, some labs report that mGluR5 activation can lead to phosphatase activation independently of PLC (Huang and Hsu, 2006; Moult et al., 2006, 2008), but the mechanism has yet to be identified.

### Experimental data on Aβ effects on synaptic plasticity

The Aβ peptide is derived from amyloid precursor protein through two stages of proteolytic cleavage and occurs in various lengths with Aβ-40 and Aβ-42 being the most common (Selkoe, 2001). Although Aβ-42 is more prone to oligomerization, both Aβ-40 and Aβ-42 can impair synaptic plasticity after they self-aggregate into oligomers of low-order (mainly dimers but possibly also oligomers of up to a dozen Aβ molecules) (Lambert et al., 1998; Walsh et al., 2002; Townsend et al., 2006; Shankar et al., 2008). For simplicity in the model, we will consider the pathogenic agent to be a low-order oligomer of Aβ without further distinction. While Aβ at high concentrations is synaptotoxic (Alberdi et al., 2010), here we focus on exposures to Aβ that impair synaptic plasticity but do not otherwise damage synapses.

Aβ oligomers suppress LTP but enhance LTD (Wang et al., 2002; Hsieh et al., 2006; Shankar et al., 2008; Li et al., 2009). Some of the mechanisms by which Aβ produces these effects on synaptic plasticity are known, and many pharmacological interventions have been suggested (Nistico et al., 2012). The mechanisms of Aβ-induced synaptic plasticity dysregulation, and putative pharmacological targets, are also diagrammed in Figure 1.

Aβ can activate α-7 nicotinic acetylcholine receptors (α7nAChRs), causing them to allow Ca to enter the spine (Wang et al., 2000; Snyder et al., 2005). This Ca entry leads to activation of PP2B, which activates STEP, which leads to reduction in NMDAR Ca conductance (Snyder et al., 2005; Shankar et al., 2008). Additionally, Aβ may increase STEP levels by inhibiting the proteosome (Shringarpure et al., 2000; Kurup et al., 2010). Presumably, these combined mechanisms cause a moderate, sustained Ca entry into the spine that promotes LTD, and cause a reduction in NMDAR Ca conductance sufficient to block LTP even with the strong, brisk presynaptic activity that otherwise would produce it. Some evidence indicates that Ca entry due to α7nAChR activation by acetylcholine (ACh) also contributes to LTP production under normal circumstances, but that this contribution is stunted by Aβ (Chen et al., 2006).

Acetylcholinesterase inhibitors improve learning and memory in a transgenic mouse AD model (Dong et al., 2005). Also, acetylcholinesterase inhibitors prevent LTP suppression due to Aβ *in vivo* in a concentration-dependent manner (high doses of acetylcholinesterase inhibitors actually cause LTP suppression in control animals) (Kapai et al., 2010, 2012). Nicotine also prevents learning and memory impairment, and LTP suppression, in an intracerebroventricular-Aβ mouse model of AD (Srivareerat et al., 2011). These observations of mostly beneficial effects of α7nAChR activation seem contradicted by the finding that transgenic AD mice that lack the gene for the α7nAChR still overexpress Aβ but retain the ability to produce LTP and have normal learning and memory capability (Dziewczapolski et al., 2009). It may be that stimulation of α7nAChRs by excess Aβ both promotes LTD and prevents LTP, but that activation of α7nAChRs by naturally occurring ACh or by nicotine is not only consistent with normal synaptic plasticity but can also normalize α7nAChR function despite exposure to excess Aβ. This possibility is suggested by the findings that the α7nAChR agonist 3-(2,4-dimethoxybenzylidene)-anabaseine (DMXB) reverses the Aβ-induced LTP deficit (Chen et al., 2006), and that the α7nAChR partial agonist 2-[2-(4-bromophenyl)-2-oxoethyl]-1-methyl pyridinium (S 24795) facilitates release of Aβ from α7nAChRs and restores normal α7nAChR function (Wang et al., 2009).

The Aβ-induced dysregulation of synaptic plasticity can be restrained through inhibition of phosphatases. Blockade of PP2B activity with FK506 or cyclosporin A prevents Aβ-induced LTP deficits and improves learning and memory in a transgenic mouse AD model (Chen et al., 2002; Dineley et al., 2007). Similarly, blockade of PP1 with tautomycin reverses LTP deficits in transgenic mouse AD models (Knobloch et al., 2007). Blockade of mGluR5s using methyl-6-(phenylethynyl) pyridine (MPEP) also prevents Aβ-induced LTP suppression (Wang et al., 2004). Note that some of these and other effects (see below in this subsection) may be protein-synthesis dependent and so not relevant to the model, which in its initial form concerns early effects that are not protein-synthesis dependent (see also Discussion).

LTP is enhanced in transgenic AD mice if they also lack STEP (Zhang et al., 2010). Resveratrol, a constituent of red wine, promotes the clearance of Aβ through a mechanism that involves activation of the proteasome (Marambaud et al., 2005). Methylene blue also reduces Aβ levels, probably via proteasome activation, and this is associated with improvement in learning and memory in a transgenic mouse AD model (Medina et al., 2011). The benefit is apparently due to reduction in Aβ, but the improvement in learning and memory also could be due to increased degradation of STEP by the activated proteasome. Aβ itself inactivates the proteasome, which causes STEP accumulation, so resveratrol or methylene blue could also reduce STEP accumulation via proteasome activation.

Aβ impairs both PI3K and Ras activation by TrkB by targeting the adaptor proteins IRS and Shc (Tong et al., 2004). This prevents suppression of GSK3 by Akt, and active GSK3 promotes LTD and prevents LTP. Inhibition of GSK3 using the specific inhibitors AR-A014418 or CT-99021 blocks Aβ-induced prevention of LTP (Jo et al., 2011; Shipton et al., 2011). Also, stimulation of TrkB receptors using BDNF or NT4 reverses Aβ-induced suppression of LTP (Zeng et al., 2010), and the small-molecule TrkB agonist 7,8-dihydroxyflavone (7,8-DHF) can reduce learning and memory deficits in a transgenic mouse AD model (Devi and Ohno, 2012).

As an alternative to blocking phosphatases, the Aβ-induced LTP deficit can also be reduced by treatments that promote kinase activity. PKA, a kinase that contributes to LTP production, is activated by cAMP, and cAMP is activated by AC (see subsection Experimental Data on Basic Mechanisms of LTP and LTD). Treatment using forskolin, which activates AC and thereby increases cAMP and PKA activation, can prevent Aβ-induced LTP suppression in the hippocampal slice (Vitolo et al., 2002). Similarly, phosphodiesterase (PDE) breaks down cAMP, and so would reduce PKA activation and also reduce its LTP contribution, but treatment using the PDE inhibitor rolipram both prevents Aβ-induced LTP suppression in the hippocampal slice and reduces LTP deficits in a transgenic mouse AD model (Vitolo et al., 2002; Gong et al., 2004). Activation of PKC using the membrane-permeable PKC agonist phorbol 12-myristate 13-acetate (PMA) also prevents the Aβ-induced LTP deficit (Zhang et al., 2009). Interestingly, inactivation of PKC using the PKC antagonist chelerythrine does not exacerbate the Aβ-induced LTP deficit.

Although Aβ oligomers bind prion protein, such binding is not involved in Aβ effects on memory (Balducci et al., 2010). In fact, recently it has been shown *in vivo* and *in vitro* that, by binding Aβ, prion protein may actually inhibit its deleterious effects on synapses (Fluharty et al., 2013). Other known interactions of Aβ with receptors or signaling molecules that are not directly relevant to early LTP and LTD production are also excluded from the model. However, even with its sharp focus, the initial model has over 50 elements, and the consequences of their interactions would be almost impossible to determine from inspection of the model diagram alone (see Figure 1). The model diagram graphically illustrates the complexity of the problem to be faced in developing pharmacological means to prevent Aβ-induced dysregulation of synaptic plasticity, and the likelihood that this complex problem will require a complex, multitarget solution has been recognized (Nistico et al., 2012). The purpose of the initial model is to begin to represent, simulate, and analyze the interactions computationally, and to initiate a more rational approach to the design of multidrug treatments for the cognitive impairment due to AD.

### Computational representation, simulation, and analysis

The interactions described above in subsections Experimental Data on Basic Mechanisms of LTP and LTD and Experimental Data on Aβ Effects on Synaptic Plasticity are represented computationally both in a Maude specification and in a MATLAB program. These two computational representations were tested for equivalence using a test battery that is a superset of the reported results, and their behavior over the whole battery is the same (see also Results). In addition to providing a crosscheck, the two representations offer distinct benefits: the Maude specification facilitated temporal-logic analysis of the model, while the MATLAB program facilitated the screening for efficacy of all possible combinations of ten pharmacological compounds.

The model contains 58 elements, each of which represents a different molecular species and is associated with a non-negative-integer level of activity that takes into account both the efficacy of individual molecules and the abundance of molecules of that species. For simplicity, many elements are restricted to levels of 0 or 1, so they are essentially binary. These integer (or in many cases binary) levels are not meant as precise measurements but have meaning in their changes relative to one another, and they reflect the statistically significant but otherwise qualitative data on which they are based. Each element is represented by an operator in Maude, which assigns to it an integer level, or simply by a variable in MATLAB. To distinguish them from actual molecules, and to emphasize that they take relative, integer levels, all model elements are rendered in monotype font using the name they are given in the computer programs. Because the programming languages do not allow Greek lettering, the variable names are different from standard abbreviations (see Table 1 for a list of abbreviations). As an example of an element/operator level assignment, Aβ at level 1 would be written as Abeta(1).

**Table 1:** **List of abbreviations**.

**Abbreviation**	**Full name**
Abeta	Amyloid-β (Aβ)
AC	Adenylyl cyclase
ACact	A compound that activates AC
ACh	Acetylcholine
AChRnorm	A compound that normalizes the function of the nAChR
Akt	Ak thymoma kinase, also known as protein kinase B
AMPAR	α-amino-3-hydroxy-5-methyl-4-isoxazole propionic acid receptor
AR1	AMPAR subunit 1
AR2	AMPAR subunit 2
BDNF	Brain-derived neurotrophic factor
Ca	Calcium
cAMP	Cyclic adenosine monophosphate
CaM	Calmodulin
CaMKII	Calcium/calmodulin-dependent protein kinase II
DAG	Diacyl glycerol
Glu	Glutamate
Gq	G-protein q
Grb2	Growth factor receptor bound-2
GSK3	Glycogen synthase kinase-3
GSK3block	A compound that blocks GSK3
I2	Inhibitor-2
IP3	Inositol trisphosphate
IP3R1	IP3 intracellular receptor 1
IRS	Insulin receptor substrate
KLC2	Kinesin light chain-2
mGluR5	Metabotropic glutamate receptor-5
mGRblock	A compound that blocks mGluR5
nAChR	Nicotinic acetylcholine receptor
NMDAR	*N*-methyl-D-aspartate receptor
NR1	NMDAR subunit 1
NR2	NMDAR subunit 2
PDE	Phosphodiesterase
PDEblock	A compound that blocks PDE
PDK1	Phosphoinositide-dependent kinase-1
PI3K	Phosphoinositide-3-kinase
PIP2	Phosphatidylinositol bisphosphate
PKA	Protein kinase A
PKC	Protein kinase C
PKCact	A compound that activates PKC
PKCblock	A compound that blocks PKC
PLC	Phospholipase C
PP1	Protein phosphatase-1
PP1block	A compound that blocks PP1
PP2B	Protein phosphatase-2B
PP2Bblock	A compound that blocks PP2B
PPI1	Phosphoprotein inhibitor-1
proACT	A compound that activates the proteosome
proteosome	Proteosome
PSD95	Post-synaptic density-95
Pyk2	Protein tyrosine kinase-2
Ras	Rat sarcoma kinase
Shc	Src homology protein
SOS	Son of sevenless
Src	Sarcoma kinase
STEP	Striatal-enriched protein tyrosine phosphatase
TARP	Transmembrane AMPAR regulatory protein
TrkB	Tyrosine kinase-B receptor
TrkBnorm	A compound that normalizes the function of TrkB

All abbreviations correspond to elements of the Aβ-synapse computer model and, for that reason, are written in monotype font.

All of the interactions in the model are based on experimental observations (see subsections Experimental Data on Basic Mechanisms of LTP and LTD and Experimental Data on Aβ Effects on Synaptic Plasticity), and some parameters (see below in this section) are set in order to bring the behavior of the model in line with empirical findings on LTP and LTD in the absence or presence of Abeta. Specifically, the model is arranged so that synaptic strength, which is represented by postsynaptic AMPAR, takes levels consistent with baseline conditions, LTP, or LTD, depending on the prevailing conditions of presynaptic activity (preSYN) and Abeta. For simplicity, presynaptic activity is limited to four levels: preSYN(0), preSYN(1), preSYN(2), and preSYN(3). Aβ is either present or absent: Abeta(0) or Abeta(1). The normal baseline AMPAR level is set to 3: AMPAR(3). Normal LTD and LTP are set, respectively, as changes down or up by 3 from baseline: AMPAR(0) or AMPAR(6). Certain pharmacological interventions can alter the AMPAR baseline (see Results), but in all cases LTD and LTP are defined respectively as decreases or increases in AMPAR level relative to baseline. Then, before any pharmacological interventions are made, the model is set as follows. In the absence of Abeta [i.e., Abeta(0)], AMPAR takes the baseline level of 3 for preSYN(0) and preSYN(1), takes the LTD level of 0 for preSYN(2), and takes the LTP level of 6 for preSYN(3). In the presence of Abeta [i.e., Abeta(1)], AMPAR also takes the baseline level of 3 for preSYN(0) but takes the LTD level of 0 for both preSYN(1) and preSYN(2) to reflect the enhancement of LTD due to Aβ, and takes the baseline level of 3 at preSYN(3) to reflect the suppression of LTP due to Aβ, as observed experimentally (see subsection Experimental Data on Aβ Effects on Synaptic Plasticity).

The rest of the Methods section is a concise but precise description of the interactions as they are implemented in the Maude and MATLAB computer programs. The detail of the description is sufficient for full program reproduction (readers not interested in this level of detail can skip the rest of Methods). The description begins with the input elements, which are those whose level is not influenced by the other (i.e., non-input) elements. Input elements include Abeta, the neurotransmitters Glu and ACh, and the neuromodulator BDNF. The levels of Glu and ACh are equal to the level of preSYN (i.e., 0, 1, 2, or 3). The BDNF level is 1 if preSYN exceeds the BDNF threshold (BDNFthr) and 0 otherwise. BDNFthr is set at 2.

For simplicity, postsynaptic depolarization is not represented explicitly but is assumed to occur for any level of preSYN greater than 0. Also for simplicity, and except for AMPAR, the level of a neurotransmitter receptor in the model represents the amount of spine Ca it admits. The level of NMDAR is equal to the sum of Glu and the levels of its subunits NR1 and NR2 except when Glu is 0, in which case NMDAR is also 0. NR1 and NR2 are both 1 but the level of NR2 is augmented to 2 if Src is greater than 0. The level of mGluR5 is 1 when Glu is greater than 0 and 0 otherwise. These levels are independent of Abeta. For Abeta(0) the level of nAChR is equal to ACh, but for Abeta(1) it is 1 when ACh is 0 and 2 when ACh is greater than 0. The neuromodulator receptor TrkB takes the same level as BDNF. Shc and IRS take the same level as TrkB, while SOS takes the same level as Shc.

Most non-input elements that receive a positive connection from only one other element simply take the same level as that other element. For example, Gq takes mGluR5, CaM takes Ca, IP3R1 takes IP3, Src takes Pyk2, and so on. Three elements receive two positive connections: PI3K takes Ras or IRS, while IP3 and DAG take PLC but only if PIP2 is 1. The level of Ca, which receives three positive connections, is simply the sum of NMDAR, nAChR, and IP3R1. The level of Pyk2, which receives a positive and a negative connection, is the difference between PKC and STEP, bounded at 0. Note that the results of all subtractions in the model are bounded at 0 so that all levels are non-negative integers. Some inhibitory elements are constitutively active at 1 unless they themselves are inhibited, in which case they are 0. Thus, PPI1 is 1 unless PKA is 0 and PP2B is 1, I2 is 1 but only if GSK3 is 0, and PP1 is 1 but only if PPI1 and I2 and CaMKII are all 0. The proteosome is 1 but only if Abeta is 0. The level of cAMP is 2 plus the difference between AC and PDE unless AC is 0, in which case cAMP is 0. PDE is 1 unless blocked by PDEblock (see below in this subsection).

The most complicated elements are GSK3, STEP, and AMPAR. GSK3 takes level 1 but this level is augmented to 2 if PP1 is 1. However, if the kinases that inactivate GSK3 are all fully active (i.e., PKA is 2, and Akt, PKC, and CaMKII are 1) then GSK3 is 0. Also, if Akt is 1 but the other kinases are not fully active then GSK3 is the sum of 1 and PP1 minus the sum of Akt and the minimum of PKA and 1. If the proteosome is 1 then STEP is PP2B minus PKA, but if the proteosome is 0 then STEP is the sum of PP2B and 1 minus PKA. Regardless of PP2B and PKA, if the proteosome is 2 then STEP is 0. The AMPAR level, which determines synaptic strength in the model, is the sum of the levels of its constituents AR1, AR2, and KLC2. AR1 is the sum of PP1 and PP2B, subtracted from the sum of 1, CaMKII, PKC, and PKC minus 1. AR2 is 1 minus STEP, while KLC2 is 2 minus GSK3. These more complicated arrangements, which are not inconsistent with observations as reported, were made to help bring the model into agreement with the data (see above in this subsection).

Bringing the model into agreement with the data was also facilitated by making several key elements binary with thresholds. Thus, PKC is 1 if Ca exceeds PKCthr. PP2B is 1 if CaM exceeds PP2Bthr, AC is 1 if CaM exceeds ACthr, and CaMKII is 1 if CaM exceeds CaMKthr (in which case PP1 is 0). Otherwise these elements are 0. The thresholds PP2Bthr, ACthr, PKCthr, and CaMKthr are set at 5, 7, 8, and 9, respectively. The thresholds were set manually in order to bring the model in line with empirical observation (see above in this subsection).

Input elements also include those representing compounds that can modify the function of receptors or signaling molecules. Consistent with findings that certain compounds can normalize nAChR function by displacing Aβ (see subsection Experimental Data on Aβ Effects on Synaptic Plasticity), AChRnorm restores the normal relationship between nAChR and ACh in the presence of Abeta in the model. Similarly, TrkBnorm restores the normal functioning of TrkB. In contrast, mGRblock is meant as a non-competitive inhibitor that simply sets mGluR5 to 0 whether Abeta is present or absent. The inhibitors GSK3block, PDEblock, PKCblock, PP1block, and PP2Bblock likewise set the level of their associated enzyme to 0. ACact sets AC to 1 under all circumstances but PKCact sets PKC to 1 only if PKCblock is 0. The proteosome is set to 2 by proACT.

The interactions described above in this subsection completely specify the Aβ-synapse model, which was implemented both as Maude and MATLAB programs. Maude and MATLAB are fundamentally different. In MATLAB, which is an imperative programming language, statements are commands that execute in strict order but in Maude, which is a declarative language, statements are descriptions of facts that execute in arbitrary order as they apply (Monin and Hinchey, 2003; Huth and Ryan, 2004; Clavel et al., 2007). To make their processing modes somewhat more similar, the interactions in the MATLAB program were expressed as conditionals within a while loop. The conditional for any element took account of the levels of all other relevant elements and set an update flag if the level of its element was updated. The while loop continued until no further updates could be made, at which point the while loop terminated. Execution terminated for all combinations of inputs, which indicates that the model as implemented in MATLAB always reached a steady-state.

The interactions in the Maude specification were expressed as a conditional declaration for each element that likewise took account of the levels of all other relevant elements, and executed only if the level of its element was changed in so doing. In Maude, declarations are either “equations” or “rules,” and all applicable equations must execute before any rule can execute (for full details see Clavel et al., 2007). In the Maude specification for the Aβ-synapse model, the declarations for STEP and NR2 were rules; all others were equations. For the purposes of temporal-logic model checking, Maude executes applicable rules in all possible orders (see also Huth and Ryan, 2004). Expressing the declarations for STEP and NR2 as rules imposed a necessary ordering (i.e., the rules went last) and facilitated checking of the Aβ-synapse model (see Results). Like the MATLAB program, the Maude specification terminated for all combinations of inputs, meaning that the model as implemented in Maude always reached a steady state. Despite the programming style adopted for the MATLAB version, the Maude and MATLAB versions of the Aβ-synapse model still employed different processing modes, yet their behavior was identical over an extensive test battery that was a superset of the results reported in the next section.

## Results

All of the results reported in this section are the same for both the MATLAB and Maude versions of the Aβ-synapse model. This crosscheck provides assurance that the reported results are free of programming error. The MATLAB and Maude versions offered distinct advantages. Specifically, the MATLAB version was used to evaluate efficiently the effects of all possible combinations of ten specific compounds on LTD and LTP in the presence of Abeta, while the Maude version was used to analyze model behavior in terms of temporal-logic.

### Model behavior under normal conditions and in the presence of Aβ

Under normal circumstances in the model [i.e., with Abeta(0)], LTD is produced at preSYN(2) and LTP is produced at preSYN(3), each by a distinct pattern of kinase/phosphatase activation and inactivation. Table 2 shows the activity of a subset of the kinases and phosphatases that are integral to the production of LTD and LTP (Table 3 includes some additional ones). Although they are under the control of a complex set of interactions, the specific kinase/phosphatase pattern that occurs at any level of presynaptic activity is determined by the level of Ca produced by that activity. With Abeta(0), AMPAR is at its baseline level of 3 both for presynaptic activity levels of 0 or 1 [preSYN(0) or preSYN(1)], and in both cases the kinase GSK3 is at its constitutive level of 1 but the kinases PKA, PKC, and CaMKII are at level 0 and the phosphatases PP2B and PP1 are likewise at level 0 (Table 2 Rows 1 and 2). With Abeta(0) and preSYN(2) the LTD pattern occurs, which is characterized by phosphatase activation and kinase inactivation except for the pro-LTD kinase GSK3, which assumes level 2 (Table 2 Row 3). With Abeta(0) and preSYN(3) the LTP pattern occurs, which is characterized by PP1 inactivation (PP2B stays active) and kinase activation (PKA at 2, PKC and CaMKII each at 1) except for GSK3, which assumes level 0 (Table 2 Row 4). These kinase/phosphatase patterns characterize normal LTD and LTP in the model.

**Table 2:** **Model element levels at different levels of presynaptic activity**.

**Row**	**preSYN**	**Abeta**	**Ca**	**PP2B**	**PP1**	**GSK3**	**PKA**	**PKC**	**CaMKII**	**NMDAR**	**AMPAR**	**Effect**
1	0	0	0	0	0	1	0	0	0	0	3	None
2	1	0	5	0	0	1	0	0	0	3	3	None
3	2	0	7	1	1	2	0	0	0	4	0	LTD
4	3	0	10	1	0	0	2	1	1	6	6	LTP
5	0	1	1	0	0	1	0	0	0	0	3	None
6	1	1	6	1	1	2	0	0	0	3	0	LTD
7	2	1	7	1	1	2	0	0	0	4	0	LTD
8	3	1	8	1	0	1	2	0	0	5	3	None

Abeta *is present for rows 1–4 but absent for rows 5–8. The same results are obtainable with the MATLAB or Maude versions of the Aβ-synapse model*.

**Table 3:** **Results of temporal-logic model checking in the absence or presence of Abeta**.

**Row**	**preSYN**	**Abeta**	**Proposition**	**Value**
1	0	0	PP2B and PP1 and STEP and PKA and PKC and CaMKII and Akt are always 0 and GSK3 is always 1	True
2	1	0	PP2B and PP1 and STEP and PKA and PKC and CaMKII and Akt are always 0 and GSK3 is always 1	True
3	2	0	AMPAR is not 0 until PP2B and PP1 and STEP are 1 and GSK3 is 2	True
4	2	0	PKA and PKC and CaMKII and Akt are always 0	True
5	3	0	AMPAR is not 6 until PKA is 2 and PKC and CaMKII and Akt are 1	True
6	3	0	PKA is always 2 and PKC and CaMKII and Akt are always 1	False
7	3	0	PKA is always 2 and PKC and Akt are always 1	True
8	3	0	CaMKII is eventually 1	True
9	3	0	CaMKII is not 1 until Src is 1	True
10	0	1	PP2B and PP1 and STEP and PKA and PKC and CaMKII and Akt are always 0 and GSK3 is always 1	True
11	1	1	PP2B and PP1 and STEP and PKA and PKC and CaMKII and Akt are always 0 and GSK3 is always 1	False
12	1	1	PP2B and PP1 are eventually 1 and GSK3 and STEP are eventually 2	True
13	3	1	PKA is eventually 2 and PKC and CaMKII and Akt are eventually 1	False
14	3	1	PKA is always 2	True
15	3	1	PKC or CaMKII or Akt are eventually 1	False

*The Maude version of the Aβ-synapse model is used for this purpose*.

In the presence of Abeta [i.e., Abeta(1)], and in the absence of any pharmacological compounds that modify receptors or signaling molecules, the LTD pattern occurs at preSYN(2) but also, aberrantly, at preSYN(1), thus enhancing LTD by expanding its range (Table 2 Rows 6 and 7). In contrast, with Abeta(1) the normal LTP pattern does not occur at preSYN(3) but the LTD pattern does not occur either. Instead, the activation of PKA thwarts the full LTD pattern and keeps the AMPAR level at baseline [i.e., AMPAR(3)], mainly through inhibition by PKA of key LTD drivers (see subsection Effects of Compounds that Modify Signaling Molecules). The kinase/phosphatase pattern that pertains with Abeta(1) and preSYN(3) appears as a hybrid between the normal LTD and LTP patterns (Table 2 Row 8). The mechanisms by which the Aβ-synapse model produces this behavior are revealed through temporal-logic model checking using the Maude specification.

The results of temporal-logic checking of the Aβ-synapse model are summarized in Table 3. In the absence of Abeta [i.e., Abeta(0)], presynaptic activity at level 1 [preSYN(1)] does not increase Ca enough to cause LTD because the phosphatases PP2B and PP1 are never activated and the pro-LTD kinase GSK3 is never raised from its constitutive level of 1 to level 2 (Table 3 Rows 1 and 2). LTD occurs (i.e., AMPAR is driven down to level 0) at presynaptic activity level 2 [preSYN(2)], but not until the phosphatases PP2B, PP1 and, additionally, STEP are activated and GSK3 attains level 2 (Table 3 Row 3). During the processes of LTD the pro-LTP kinases PKA, PKC, CaMKII and, additionally, Akt are never activated (Table 3 Row 4).

Again without Abeta [i.e., Abeta(0)], LTP occurs (i.e., AMPAR is brought up to level 6) at preSYN(3) but not until the kinase PKA reaches level 2 and the other pro-LTP kinases PKC, CaMKII, and Akt are active at level 1 (Table 3 Row 5). Further analysis shows that this is not always the case for Abeta(0) and preSYN(3), but that activation of CaMKII requires prior activation of Src (Table 3 Rows 6–9). This indicates that the Ca level does not cross the CaMKII threshold until Src is activated. Thus, closure of the Src loop, with increased Ca influx through the NMDAR, is critical for full LTP expression in the model.

At the presynaptic activity level of 0 [preSYN(0)] the kinase/phosphatase pattern is the same in the presence or absence of Abeta (Table 3 Rows 10 and 1), but LTD occurs aberrantly at preSYN(1) with Abeta(1) because Ca is high enough to bring the phosphatases PP2B, PP1, and STEP, and the kinase GSK3, into the LTD pattern (Table 3 Rows 11 and 12). In contrast, with Abeta(1), presynaptic activity at level 3 [i.e., preSYN(3)] is not sufficient to raise Ca enough to elicit the full LTP kinase activation pattern (Table 3 Row 13). However, with Abeta(1), preSYN(3) does raise Ca high enough to bring PKA to level 2 (Table 3 Row 14), and this thwarts LTD through inactivation of PP1 and STEP and return of GSK3 to its constitutive level of 1 by the actions of PKA (see subsection Effects of Compounds that Modify Signaling Molecules). Still, the rise in Ca with Abeta(1) and preSYN(3) is not high enough to activate any of the other kinases in addition to PKA that are necessary for LTP (Table 3 Row 15).

### Effects of compounds that modify receptor function

Dysregulation of synaptic plasticity due to Abeta occurs primarily because Abeta alters Ca influx through the nAChR and also blocks signal transduction by the TrkB receptor in the model. Therefore, compounds that restore normal receptor functioning in the model can offset the dysregulatory effects of Abeta. In the presence of Abeta [i.e., Abeta(1)], AChRnorm by itself normalizes LTD and almost fully restores LTP (Table 4 Row 2). It does this because it normalizes Ca levels at all preSYN levels. At preSYN(3) with AChRnorm(1), PKA and PKC are always active and CaMKII eventually becomes active. However, Abeta(1) still causes TrkB signaling failure, with consequent failure to activate Akt and failure to inactivate GSK3. LTP is not fully restored because GSK3 is still able to reduce the AMPAR level through impairment of the normal function of KLC2. Administration of AChRnorm in combination with TrkBnorm rectifies this situation and fully restores LTP while also normalizing LTD (Table 4 Row 6). TrkBnorm by itself does not diminish the LTD enhancement due to Abeta(1) but it partially restores LTP at preSYN(3) by allowing TrkB activation by BDNF and consequent activation of Akt and inactivation of GSK3 (Table 4 Row 4).

**Table 4:** **Effects on synaptic plasticity of compounds that modify receptor function**.

**Row**	**preSYN**	**AChRnorm**	**mGRblock**	**TrkBnorm**	**AMPAR**	**Effect**
1	(0, 1, 2, 3)	0	0	0	(3, 0, 0, 3)	Abeta dysregulation: LTD enhanced, LTP eliminated
2	(0, 1, 2, 3)	1	0	0	(3, 3, 0, 5)	LTD normalized, LTP largely restored
3	(0, 1, 2, 3)	0	1	0	(3, 3, 0, 0)	LTD normalized, LTP is reversed to LTD
4	(0, 1, 2, 3)	0	0	1	(3, 0, 0, 4)	LTD still enhanced, LTP partially restored
5	(0, 1, 2, 3)	1	1	0	(3, 3, 0, 3)	LTD normalized, LTP still eliminated
6	(0, 1, 2, 3)	1	0	1	(3, 3, 0, 6)	LTD normalized, LTP fully restored
7	(0, 1, 2, 3)	0	1	1	(3, 3, 0, 1)	LTD normalized, LTP partially reversed to LTD
8	(0, 1, 2, 3)	1	1	1	(3, 3, 0, 4)	LTD normalized, LTP partially restored

*Abeta(1) pertains in all cases. Row 1 reiterates the unaltered Abeta effects from Table 2 Rows 5–8. None of the combinations alter the baseline AMPAR level. The same results are obtainable with the MATLAB or Maude versions of the Aβ-synapse model*.

mGRblock by itself with Abeta(1) normalizes LTD but converts LTP to a full LTD (Table 4 Row 3). It does this because it blocks the mGluR5-induced opening of intracellular Ca stores and reduces Ca by 1 at all levels of preSYN except preSYN(0), at which level mGluR5 would not be active anyway. The reduction by 1 of Ca level due to mGRblock prevents the pattern of activation of PP2B, PP1, STEP, and GSK3 that supports LTD at preSYN(1) but allows it at preSYN(2) (see also Table 3). At preSYN(3) the reduction in Ca level due to mGRblock exacerbates the reduction due to Abeta, which is already enough to prevent the pattern of activation of PKA, PKC, CaMKII, and Akt that supports LTP. Furthermore, reduction of the Ca level due to mGRblock also prevents the activation of PKA that would otherwise thwart LTD at preSYN(3) in the presence of Abeta (Table 2 Row 8), and allows the kinase/phosphatase pattern that supports LTD instead. Thus mGRblock does not reduce the expansion of the LTD range due to Abeta but pushes it to a higher level of presynaptic activity.

Combined administration of mGRblock and AChRnorm together with Abeta(1) (Table 4 Row 5) produces less normalization of synaptic plasticity than administration of AChRnorm alone (Table 4 Row 2) because mGRblock lowers Ca enough at preSYN(3) to prevent LTP. However, the extension of LTD to preSYN(3) due to mGRblock alone (Table 4 Row 3) is prevented by AChRnorm when combined with mGRblock (Table 4 Row 5). This occurs at preSYN(3) because the Ca level with AChRnorm(1) and mGRblock(1) is not enough to activate PKC or CaMKII but is enough to activate PKA, and this effectively cancels LTD (see next subsection). As explained above, TrkB activation due to TrkBnorm by itself can augment LTP by 1, and it also does so in conjunction with mGRblock (Table 4 Row 7) or with AChRnorm and mGRblock (Table 4 Row 8).

### Effects of compounds that modify signaling molecules

**Figure 2:**
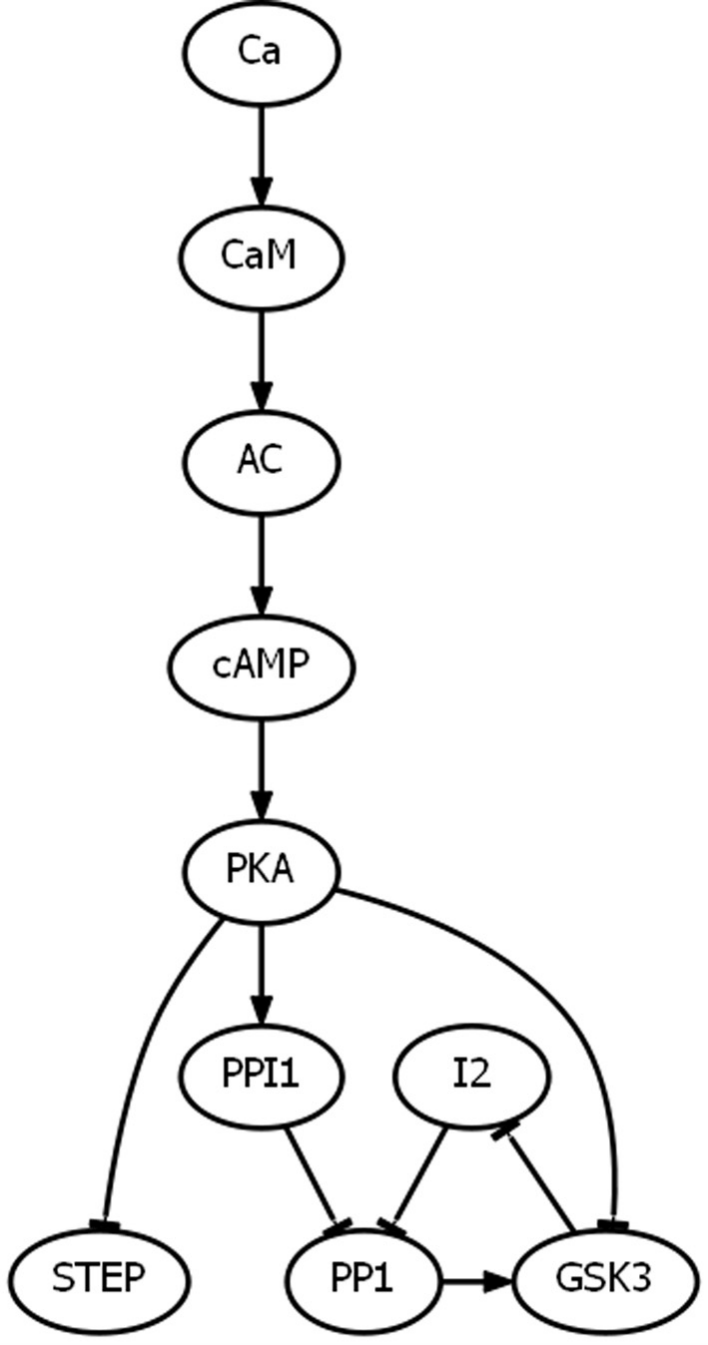
**The subset of interactions involved in PKA suppression of LTD**. Experiments suggest (see subsection Experimental Data on Basic Mechanisms of LTP and LTD) that AC has a relatively high threshold and is only activated (via CaM) at the high levels of Ca that produce LTP. Once activated (via AC and cAMP), PKA suppresses LTD by inhibiting STEP, PP1, and GSK3. PKA inhibits STEP and GSK3 directly, but inhibits PP1 indirectly by activating PPI1 and by inhibiting GSK3, which would otherwise activate PP1 via disinhibition through I2.

The effect of PKCblock with Abeta(0) (i.e., in the absence of Abeta) and with preSYN at (0, 1, 2, 3) is AMPAR at (3, 3, 0, 4), which is normal LTD but impaired LTP. So PKC makes a substantial contribution to LTP in the model but does nothing to hinder LTD. The effect of PKCblock with Abeta(1) (i.e., in the presence of Abeta) and with preSYN at (0, 1, 2, 3) is AMPAR at (3, 0, 0, 3), which is no change in the synaptic plasticity dysregulation due to Abeta. Thus, blocking PKC does not improve plasticity, but it does not exacerbate the Abeta impairment of LTP either. This reproduces experimental findings and suggests that PKA, rather than PKC, is what keeps the synapse at least at baseline in the presence of Aβ at high levels of presynaptic activity and prevents the further expansion of LTD into the presynaptic-activity range that normally evokes LTP. More generally, the model suggests that PKA plays a central role in synaptic plasticity by allowing LTD as Ca is increased, but then by blocking LTD and allowing LTP as Ca is further increased. The mechanism may involve AC, which is activated by Ca via CaM and then activates PKA via cAMP. If the activation threshold for AC is relatively high, as experimental data suggests it is (Kim et al., 2010), then AC would only activate PKA at the high Ca levels that should normally evoke LTP, after which PKA would inhibit STEP, PP1, and GSK3, which are critical drivers of LTD. The subset of interactions involved in LTD suppression by PKA is highlighted in Figure 2 (see also Discussion).

ACact does not improve LTP by itself, nor is there any combination of ACact with other signaling compounds that improves LTP with Abeta(1). The effect of ACact in the model is to ensure that AC is active even if CaM is below ACthr, and this maintains cAMP at a high level and ensures that PKA is always active. In the model, PKA plays a critical role by canceling LTD at preSYN(3) with Abeta(1). By keeping PKA always active, ACact reduces LTD at preSYN(1), which moves plasticity toward normal, but impairs LTD at preSYN(2), which is abnormal. ACact administered by itself in the model does not prevent the LTP suppression due to Abeta at preSYN(3). This makes AC an unsuitable target for normalizing synaptic plasticity with Abeta(1) in the model. PDEblock boosts PKA activity more flexibly because it does so only when CaM is above ACthr, and PDE seems to be a better drug target than AC for normalization of synaptic plasticity (see below in this subsection).

PP2Bblock by itself and several combinations of PP2Bblock with other signaling compounds improve LTP, but all of them do so by completely eliminating LTD at preSYN(1) and preSYN(2) and some even cause a small LTP at preSYN(2). In the model, PP2B plays a critical role by supporting LTD, but this also makes PP2B an unsuitable target for normalizing synaptic plasticity in the presence of Abeta. Unlike both ACact and PP2Bblock, there are several combinations of GSK3block and other signaling compounds that can improve LTP and reduce LTD enhancement, either by reducing the amount of LTD equally at both preSYN(1) and preSYN(2), or by shifting LTD to a lower level (see below in this subsection). However, in all cases the inclusion of GSK3block in the mix increases AMPAR baseline but does not increase the relative, normalizing effects on LTP or LTD over that which can be achieved using other combinations of compounds.

With AC, PP2B, and GSK3 excluded as drug targets, the model suggests that PDE, PKC, PP1, and the proteosome might be more suitable as drug targets for the normalization of synaptic plasticity in the presence of Aβ. Table 5 lists some combinations of signaling molecule modification compounds and their effects on synaptic plasticity with Abeta(1) in the model.

**Table 5:** **Effects on synaptic plasticity of compounds that modify signaling molecules**.

**Row**	**preSYN**	**PDEblock**	**PKCact**	**PP1block**	**proACT**	**AMPAR**	**Effect**
1	(0, 1, 2, 3)	0	0	0	0	(3, 0, 0, 3)	Abeta dysregulation:
							LTD enhanced (and range expanded)
							LTP eliminated (suppressed)
2	(0, 1, 2, 3)	1	0	0	0	(3, 0, 0, 4)	LTD enhancement unchanged
							LTP partially restored
3	(0, 1, 2, 3)	1	0	0	1	(3, 1, 1, 4)	LTD enhancement reduced
							LTP partially restored
4	(0, 1, 2, 3)	1	0	1	0	(3, 1, 1, 4)	LTD enhancement reduced
							LTP partially restored
5	(0, 1, 2, 3)	1	0	1	1	(3, 2, 2, 4)	LTD enhancement further reduced
							LTP partially restored
6	(0, 1, 2, 3)	1	1	0	1	(4, 1, 5, 5)	AMPAR baseline increased
							LTD shifted to lower level
							LTP partially restored but LTP range expanded
7	(0, 1, 2, 3)	1	1	1	1	(4, 3, 5, 5)	AMPAR baseline increased
							LTD shifted to lower level and reduced
							LTP partially restored but LTP range expanded

*Abeta(1) pertains in all cases. Row 1 reiterates the unaltered Abeta effects from Table 2 Rows 5–8. The same results are obtainable with the MATLAB or Maude versions of the model*.

With Abeta(1), PDEblock by itself does not reduce the LTD enhancement but it does slightly improve LTP (Table 5 Row 2). PDEblock does not alter the Ca level at any level of preSYN, so the behavior of the model with Abeta(1) is largely the same with PDEblock as without it. Specifically, the kinases and phosphatases take the LTD pattern at preSYN(1) and preSYN(2), and PKA is activated and thwarts LTD at preSYN(3), whether PDEblock is present or absent. But PDEblock boosts PKA by 1, selectively when Ca surpasses the AC threshold (ACthr), and this increases the PKA contribution to the AMPAR level at preSYN(3), thus providing some improvement in LTP.

Combining PDEblock and proACT with Abeta(1) adds some reduction in LTD enhancement to the improvement in LTP due to PDEblock (Table 5 Row 3). With or without PDEblock, as explained above, PKA is activated at preSYN(3) and thwarts LTD. PKA does this in part by suppressing STEP which, among other functions, reduces the AMPAR level by 1. The added benefit of proACT, which stimulates the proteosome, is that it suppresses STEP at preSYN(1) and preSYN(2), thereby boosting AMPAR by 1 at those levels. This does not reduce the range of LTD with Abeta(1) but it does reduce the overall amount of LTD, and this could provide some benefit at the behavioral level (see Discussion).

Combining PDEblock and PP1block with Abeta(1) adds the same reduction in LTD enhancement to the improvement in LTP due to PDEblock (Table 5 Row 4), but it does that through a different mechanism. PP1, among other functions, boosts GSK3 by 1, and GSK3, like STEP, also reduces the AMPAR level. By blocking PP1, PP1block also reduces the GSK3 level by 1 and this increases the AMPAR level by 1 at preSYN(1) and preSYN(2). Crucially, proACT and PP1block increase AMPAR through different mechanisms: proACT suppresses STEP thereby increasing the AR2 subunit of AMPAR by 1, while PP1block suppresses GSK3 thereby increasing the KLC2 component of AMPAR by 1. Because these factors work through different pathways their effects do not occlude one another but sum together, and combining PDEblock with both proACT and PP1block further reduces the LTD enhancement at preSYN(1) and preSYN(2) (Table 5 Row 5).

With Abeta(1), combining PDEblock with proACT and PKCact increases the AMPAR baseline, but it provides the potentially beneficial effects of reducing the range of LTD while increasing both the level and range of LTP (Table 5 Row 6). By keeping PKC active, a side effect of PKCact is to raise the AMPAR baseline because, among other functions, PKC directly increases the AMPAR level by increasing the AR1 subunit level. This contribution is cancelled when PP1 is active, which occurs at preSYN(1) with this combination (PDEblock, proACT, and PKCact). The main effect of PKCact in conjunction with proACT is to increase the Ca level by 1 at all levels of preSYN greater than 0. This occurs because PKC activates Pyk2, while the proteosome inactivates STEP, and those effects together activate Src, which increases the NMDAR conductance for Ca. That does not appreciably change the behavior of the model at preSYN(1), and the LTD kinase/phosphatase pattern predominates there except that STEP is 0 due to proACT. However, at preSYN(2) the Ca level is now sufficient to cross the AC threshold and this activates PKA, which attains a higher level than normal because of PDEblock. This promotes LTP. The same occurs at preSYN(3) because of PDEblock (see above in this subsection for effects of PDEblock).

Combining PDEblock with proACT, PKCact, and PP1block also raises the AMPAR baseline and increases both the level and range of LTP, but it reduces LTD at preSYN(1) (Table 5 Row 7). It does this in two ways: it suppresses GSK3 thereby increasing the KLC2 component of AMPAR by 1 (see above in this subsection for effects of PP1block), and simply by blocking PP1 it prevents PP1 from canceling the PKC contribution to the AR1 subunit that is brought about through PKCact. With proACT suppressing STEP and increasing the AR2 subunit by 1 (see above in this subsection for effects of proACT), the net activation of AMPAR at preSYN(1) is 3. While this combination does not return synaptic plasticity to normal, these compound effects may offer some benefit in terms of reducing the deleterious consequences of Aβ for synaptic plasticity at the behavioral level (see Discussion).

### Effects of compounds that target receptors and signaling molecules together

In the presence of Abeta [i.e., Abeta(1)], using AChRnorm by itself normalizes LTD and almost fully restores LTP (Table 4 Row 2). Using AChRnorm in conjunction with PDEblock provides full normalization of LTD and full restoration of LTP with no change in AMPAR baseline (perfect plasticity; Table 6 Row 2). This occurs because PDEblock boosts PKA, and so also boosts the contribution of PKA to the AMPAR level, but only after AC is activated at preSYN(3) and generates cAMP. Many other combinations of AChRnorm and signaling compounds can also change synaptic plasticity with Abeta(1) but no other such combination (besides AChRnorm and PDEblock) also achieves perfect plasticity.

**Table 6:** **Effects on synaptic plasticity of compounds that modify receptors or signalers**.

**Row**	**preSYN**	**AChRnorm**	**mGRblock**	**TrkBnorm**	**PDEblock**	**PKCact**	**PP1block**	**proACT**	**AMPAR**	**Effect**
1	(0, 1, 2, 3)	0	0	0	0	0	0	0	(3, 0, 0, 3)	Abeta dysregulation:
										LTD range expanded
										LTP eliminated
2	(0, 1, 2, 3)	1	0	0	1	0	0	0	(3, 3, 0, 6)	LTD normalized
										LTP fully restored
3	(0, 1, 2, 3)	0	1	0	1	1	0	1	(4, 1, 1, 5)	AMPAR baseline increased
										LTD enhancement unchanged
										LTP partially restored
4	(0, 1, 2, 3)	0	1	0	1	1	1	1	(4, 3, 3, 5)	AMPAR baseline increased
										LTD enhancement reduced
										LTP partially restored
5	(0, 1, 2, 3)	0	0	1	0	0	1	1	(3, 2, 2, 4)	LTD enhancement reduced
										LTP partially restored
6	(0, 1, 2, 3)	0	0	1	1	0	1	1	(3, 2, 2, 5)	LTD enhancement reduced
										LTP further restored
7	(0, 1, 2, 3)	0	0	1	0	1	0	1	(4, 1, 4, 5)	AMPAR baseline increased
										LTD shifted to lower level
										LTP partially restored
8	(0, 1, 2, 3)	0	0	1	0	1	1	1	(4, 3, 4, 5)	AMPAR baseline increased
										LTD shifted and reduced
										LTP partially restored

*Abeta(1) pertains in all cases. Row 1 reiterates the unaltered Abeta effects from Table 2 Rows 5–8. The same results are obtainable with the MATLAB or Maude versions of the model*.

Using mGRblock by itself with Abeta(1) prevents LTD at preSYN(1) but allows it at preSYN(2) and, aberrantly, allows LTD also at preSYN(3) (Table 4 Row 3). There is no compound for modification of signaling molecules in the model that in conjunction with mGRblock gives normal AMPAR baseline with normal LTD [i.e., LTD only at preSYN(2)], and with LTP greater than AMPAR baseline at preSYN(3). Some combinations provide improvement in LTP but at the expense of baseline increase and LTD dysregulation. Two noteworthy examples involve combinations of signaling modifiers that already improve synaptic plasticity without mGRblock. Combining PDEblock, PKCact, and proACT raises the AMPAR baseline but produces a small, net LTP improvement at preSYN(3). This combination produces no net reduction in LTD at preSYN(1) but at preSYN(2) it converts LTD to LTP (Table 5 Row 6). Combining PDEblock, PKCact, proACT, and mGRblock (i.e., adding mGRblock to the mix) converts plasticity at preSYN(2) back to LTD, thereby restricting LTP to preSYN(3) (Table 6 Row 3). The same phenomenon occurs for the combination PDEblock, PKCact, proACT, and PP1block, which again provides net improvement in LTP at preSYN(3) despite raising AMPAR baseline but also causes LTP to occur, aberrantly, at preSYN(2) (Table 5 Row 7). Of potential benefit (see Discussion), this combination reduces the amount of LTD at preSYN(1) (again Table 5 Row 7). As in the previous case (Table 6 Row 3), the effect of adding mGRblock to this combination is to convert LTP back to LTD at preSYN(2), thereby restricting LTP to preSYN(3) (Table 6 Row 4). mGRblock achieves this effect by lowering Ca at all preSYN levels greater than 0 (see subsection Effects of Compounds that Modify Receptor Function). Other combinations of mGRblock and signaling molecule modification compounds achieve the same reduction in LTD enhancement and improvement of LTP but further raise AMPAR baseline.

With Abeta(1), using TrkBnorm by itself provides a slight improvement in LTP but does not diminish LTD enhancement (Table 4 Row 4). As with mGRblock, there is no combination of TrkBnorm with signaling modification compounds that gives more LTD for preSYN(2) than for preSYN(1) and gives any improvement in LTP at preSYN(3). However, by boosting the AMPAR level at preSYN(3), TrkBnorm can also improve LTP and reduce LTD enhancement in combination with signaling molecule modifiers. By themselves, PP1block or proACT reduce the LTD enhancement due to Abeta by reducing the reduction in AMPAR level each by 1 at preSYN(1) and preSYN(2). Combining TrkBnorm with PP1block and proACT provides the same incremental LTP improvement as TrkBnorm alone and also sums the reductions in LTD enhancement due to PP1block and proACT (Table 6 Row 5). The combination PDEblock, proACT, and PP1block (Table 5 Row 5) produces the same change in plasticity (i.e., slight LTP improvement and reduction of LTD enhancement) as the combination TrkBnorm, PP1block, and proACT (Table 6 Row 5), and the union of these combinations, which is TrkBnorm, PDEblock, PP1block, and proACT, achieves the same LTD reduction with more improvement in LTP (Table 6 Row 6). Other combinations of TrkBnorm with signaling modifiers further raise AMPAR baseline but, relative to that baseline, do not give better reduction of LTD enhancement or better LTP improvement.

As explained in subsection Effects of Compounds that Modify Signaling Molecules, the main effect of the combination of PKCact and proACT with Abeta(1) is to increase the Ca level at all levels of preSYN greater than 0. Another benefit of proACT is inactivation of STEP, while a side effect of PKCact is augmentation of the AMPAR baseline. The upshot of this is restriction of LTD to preSYN(1), with an augmented AMPAR baseline at all other preSYN levels. Adding TrkBnorm to this duo, yielding the combination TrkBnorm, PKCact, and proACT, adds a slight LTP improvement at preSYN(3) (Table 6 Row 7). The combination PKCact, proACT, and PP1block produces effects similar to PKCact and proACT but, by suppressing PP1, PP1block reduces the amount of LTD that is limited by the other two factors to preSYN(1). Now adding TrkBnorm to this trio, yielding the combination TrkBnorm, PKCact, proACT, and PP1block, adds an incremental LTP improvement at preSYN(3) (Table 6 Row 8). While these behaviors are far from normal, they at least move the dysregulated plasticity due to Abeta back toward normal and in ways that could be therapeutically significant (see Discussion).

## Discussion

The main purpose of the model is to suggest combinations of compounds that could be tested for efficacy in normalizing synaptic plasticity in the presence of Aβ. Validated combinations could be considered for development as pharmaceutical treatments. An ancillary purpose is to provide a view of the operation of the molecular machinery of the synapse and how that is affected by Aβ. For example, in the normal synapse (i.e., in the absence of Aβ) the model suggests that PKA is responsible for keeping STEP (and other key LTD drivers) inactive when Ca is high enough to elicit LTP (see Figure 2). Exploration of this interaction could further elucidate the role of STEP in overall synaptic function (Moult et al., 2006; Zhang et al., 2008). In the diseased synapse (i.e., in the presence of Aβ), the model suggests that the action of PKA is instrumental in preventing LTD from occurring at all non-zero levels of presynaptic activity including that which would evoke LTP in the normal synapse. The model further suggests that PKA is the mediator that keeps the diseased synapse at least at baseline at high levels of presynaptic activity.

The model provides an initial framework in which to understand how various drugs and drug combinations might operate in the diseased synapse. The model suggests that normalization of nAChR function may be the most effective way to counteract the adverse effects of Aβ on synaptic plasticity, lending some modeling support to the suggestion that disordered nAChR function is the main route by which Aβ dysregulates synaptic plasticity (Wang et al., 2000; Snyder et al., 2005; Shankar et al., 2008). Some studies suggest that certain compounds, such as S 24795, can actually displace Aβ from the nAChR and restore its normal function (Chen et al., 2006; Wang et al., 2009). The model identifies these compounds as exciting candidates for further drug development, either alone or in combination with other compounds such as phosphodiesterase inhibitors.

The Aβ-synapse model is based on findings from the literature, mainly from *in vitro* experiments using brain slice preparations. Overall model behavior is consistent with the observation from whole animals and humans that Aβ overproduction leads to cognitive impairment and specifically to deficits in learning and memory. As a data-driven model, the Aβ-synapse model does not rely on unproven assumptions, and it is readily testable using the same techniques that were used to gather the data on which the model is based (see below in this section). Of course, the Aβ-synapse model is also limited by the available data, which is incomplete, obtained using diverse and unstandardized preparations, and in a very few cases may in fact be wrong. Yet researchers have no option other than to generate their hypotheses on the basis of available data. The main advantage of the model is to gather together, in a precisely defined manner, a much larger set of data than possibly could be held in mind by individual researchers, and to determine the aggregate implications of those data through computational simulations and analyses. The result is a large number of experimentally testable predictions that are based on much of what is currently known concerning the adverse effects of Aβ on synaptic plasticity.

Most *in vitro* studies of Aβ-induced dysregulation of synaptic plasticity concern only one mechanism and focus on LTP suppression without regard for the LTD enhancement that is also induced by Aβ (see subsection Experimental Data on Aβ Effects on Synaptic Plasticity). The Aβ-synapse model takes a much broader view and considers many mechanisms, and the effects of many compounds, on both LTP and LTD. The model represents the results of many labs, each using different preparations, and it does not conform in detail to any one preparation. Instead, the model adopts a general framework in anticipation of the standardization of preparations that will be required for the achievement of a global understanding of the effects of Aβ on synaptic plasticity.

Even in its initial form, the model offers a perspective on potential multidrug therapies that could protect synapses from the adverse effects of Aβ. For example, many individual compounds that improve LTP *in vitro* also improve learning and memory *in vivo* (see subsection Experimental Data on Aβ Effects on Synaptic Plasticity). The model suggests that some of these single compounds may not also reduce Aβ-induced LTD enhancement (see Results), in which case the LTP increase alone is enough to cause the observed benefit on the behavioral level. The model suggests many combinations that may be more effective than single compounds in normalizing the plasticity of the AD synapse (see Results), and it is reasonable to suppose that they may provide further behavioral benefit as well.

One aim of this initial model is to provide a framework that could be expanded into a comprehensive view of the AD synapse. An obvious but challenging area of expansion is into mechanisms of LTP and LTD that involve new protein synthesis. Other important expansion areas include those that address practical aspects of drug design such as the relative efficacies and pharmacokinetics of specific compounds, and issues specific to human biology and pharmacology. The data-driven Aβ-synapse model is expandable by design, because it is based not on preconceived ideas but directly on data concerning the interactions among molecular species and subcellular organelles as determined experimentally. Because the model is based on data that are almost entirely qualitative, elements in the model are restricted to take one of a few integer levels that reflect relative increases or decreases in activity (see subsection Computational Representation, Simulation, and Analysis). Because of the discrete nature of the model, changes in parameters such as thresholds would have drastic effects and would cause model behavior to disagree with observation. However, the model parameters that influence the interactions, as well as the specifications of the interactions themselves, are all subject to revision on the basis of the results of tests of model predictions.

Another aim of the initial model is to initiate a computational/experimental interaction in which model predictions are tested by an array of experimentalists working in different labs (but perhaps eventually using a standardized preparation). The test results would be used to correct or confirm, refine, and expand the model, the model would be used to generate new predictions, and that cycle would continue, producing a model of ever increasing explanatory and predictive power. Most of the data for the initial Aβ-synapse model was derived from *in vitro* experiments using brain slice preparations, and the predictions of the initial model would be most effectively tested using the brain slice. It would also be reasonable to start with a standard preparation in which protein synthesis is inhibited, and then add protein synthesis mechanisms after the model and the data achieve complete agreement without them. There is no *a priori* reason to expect any limitations on the growth of the model. Even with its focus trained on the synaptic level, it should be possible to expand the model by incorporating enough extra-synaptic influences such as levels of neuromodulators, hormones, and cytokines, so that model predictions will be relevant on the whole-organism level and can be tested on that level.

The Aβ-synapse model is readily expandable, fully adjustable, and highly testable. Every modeling result is essentially a modeling prediction, and every experimental result that is not confirmatory can be used to correct the model. As such the Aβ-synapse model will serve as a valuable adjunct to experiment in the search for treatments for AD, but it will also have value in its own right as a conceptual tool. By combining imperative (i.e., MATLAB) and declarative (i.e., Maude) computer modeling modalities, the initial Aβ-synapse model illustrates how complex neurobiological and neuropathological processes can be represented, simulated, and analyzed computationally to gain deeper understanding of basic mechanisms, new rationales for multitarget drug design, and new insights into possible treatments.

## Author contributions

Thomas J. Anastasio conceived the study, reviewed the literature, designed the computational model, wrote the computer programs, performed all computational simulations and analyses, interpreted the results of all computer simulations and analyses, made all the figures and tables, wrote and approved the manuscript, and is accountable for all aspects of the work. The author emphasizes that all of the results of this computational study are *predictions* that need to be verified experimentally before they could be used in clinical trials.

### Conflict of interest statement

The author declares that the research was conducted in the absence of any commercial or financial relationships that could be construed as a potential conflict of interest.
